# Cortical response characteristics of passive, active, and resistance movements: a multi-channel fNRIS study

**DOI:** 10.3389/fnhum.2024.1419140

**Published:** 2024-08-14

**Authors:** Wenxi Li, Guangyue Zhu, Yichen Jiang, Cheng Miao, Guohui Zhang, Dongsheng Xu

**Affiliations:** ^1^Department of Rehabilitation Medicine, Yueyang Hospital of Integrated Traditional Chinese and Western Medicine, Shanghai University of Traditional Chinese Medicine, Shanghai, China; ^2^Department of Rehabilitation, Shanghai University of Traditional Chinese Medicine, Shanghai, China; ^3^Engineering Research Center of Traditional Chinese Medicine Intelligent Rehabilitation, Ministry of Education, Shanghai, China

**Keywords:** sensorimotor function, active movement, passive movement, resistance movement, cortex, fNIRS

## Abstract

**Objective:**

This study aimed to explore the impact of exercise training modes on sensory and motor-related cortex excitability using functional near-infrared spectroscopy technology (fNIRS) and reveal specific cortical effects.

**Materials and methods:**

Twenty participants with no known health conditions took part in a study involving passive, active, and resistance tasks facilitated by an upper-limb robot, using a block design. The participants wore functional near-infrared spectroscopy (fNIRS) devices throughout the experiment to monitor changes in cortical blood oxygen levels during the tasks. The fNIRS optode coverage primarily targeted key areas of the brain cortex, including the primary motor cortex (M1), primary somatosensory cortex (S1), supplementary motor area (SMA), and premotor cortex (PMC) on both hemispheres. The study evaluated cortical activation areas, intensity, and lateralization values.

**Results:**

Passive movement primarily activates M1 and part of S1, while active movement mainly activates contralateral M1 and S1. Resistance training activates brain regions in both hemispheres, including contralateral M1, S1, SMA, and PMC, as well as ipsilateral M1, S1, SMA, and PMC. Resistance movement also activates the ipsilateral sensorimotor cortex (S1, SMA, PMC) more than active or passive movement. Active movement has higher contralateral activation in M1 compared to passive movement. Resistance and active movements increase brain activity more than passive movement. Different movements activate various cortical areas equally on both sides, but lateralization differs. The correlation between lateralization of brain regions is significant in the right cortex but not in the left cortex during three movement patterns.

**Conclusion:**

All types of exercise boost motor cortex excitability, but resistance exercise activates both sides of the motor cortex more extensively. The PMC is crucial for intense workouts. The right cortex shows better coordination during motor tasks than the left. fNIRS findings can help determine the length of treatment sessions.

## 1 Introduction

Motor function is an essential ability for human production and life (Hu et al., 2022). When partially or completely absent, it can lead to significant limitations in an individual’s daily activities. Exercise therapy is typically used as an important tool to help patients with motor dysfunction (e.g., fractures, Parkinson’s disease, stroke) regain physical function. This form of therapy is a fundamental aspect of physical rehabilitation (Smidt et al., 2005; Lee et al., 2022).

Exercise therapy can be categorized into three main types based on movement initiation: passive, active, and resistance exercise (Lindberg et al., 2004). Passive exercise relies entirely on external forces, like equipment, assistance from others, or even using one’s healthy limbs, to complete the movement (Chiyohara et al., 2020) and is primarily used to restore and maintain joint mobility (Trinity and Richardson, 2019). In contrast, active exercise requires the patient to perform the movement independently (Kai et al., 2016), making it the most common form of training in clinical practice. This type of exercise aims to increase muscle strength, endurance through specific tasks (Kwakkel et al., 2015). It is now understood that resistance training, suitable for individuals with at least grade 3 muscle strength on a manual muscle test (MMT), tailors resistance to their tolerance (Veldema and Jansen, 2020) and effectively maintains muscle size and endurance (Shi et al., 2021). During rehabilitation, therapists select the most suitable training mode based on individual needs. For example, in stroke rehabilitation, passive training helps patients regain movement in paralyzed limbs during the early stages. As function improves, active exercise becomes the mainstay for enhancing motor function in the affected limb (Hsu et al., 2022). Finally, if active movement is possible, resistance training is gradually incorporated to build upper limb strength and endurance, allowing patients to tackle more complex tasks (Ghous et al., 2017). Physical exercise training demonstrably improves brain plasticity, crucial for functional connection and reorganization of the damaged cortex in stroke patients, promoting motor function recovery (Kokotilo et al., 2009; James and McGlinchey, 2022). While all three movement patterns activate key areas of the cerebral cortex associated with movement (S1, M1, PMC, and SMA), they differ in activation level, range, and mechanisms (Chye et al., 2010; Chang, 2017). Compared to passive movements, which primarily activate M1 through sensory input (touch, proprioception), active movements engage the same areas but with greater cortical activation (Pittaccio et al., 2013). Passive movement emphasizes the importance of sensory input (Hosseini et al., 2019); it activates M1 either directly through S1 by sensory inputs (touch, proprioception) or indirectly through modulation of SMA by S1. In contrast, during active movement, the SMA, which is involved in both planning and executing movements, is typically activated first. Subsequently, it influences the S1 and M1 areas (Nasrallah et al., 2019). During resistance training, the level of subject involvement from the patient is typically higher compared to other forms of exercise therapy, resulting in a greater exercise load and a need for stronger motor control and coordination (Park et al., 2019). As a result, a larger sensory motor area is engaged, leading to more significant activation (Shi et al., 2021). Furthermore, the neural adaptations resulting from resistance training occur not only in the cerebral cortex but also in the reticulospinal tract, causing changes in synaptic efficiency between motor neurons or interneurons and achieving an increase in strength (Škarabot et al., 2021). The effectiveness of exercise training is influenced by a combination of factors, including type, dosage, and the progression of exercise (Donnellan-Fernandez et al., 2022). During stroke rehabilitation, therapists leverage their expertise to tailor training modes and tasks based on individual needs, often employing all three modes at different stages of the recovery process (Doumen et al., 2023). However, the effects of different movement patterns on somatosensory cortex reorganization in each stage of stroke rehabilitation remain unknown. This lack of knowledge hinders the design of truly effective clinical treatment plans. Additionally, existing research does not provide specific guidance on determining the optimal dosage and intensity of exercise for maximizing rehabilitation outcomes, considering both time efficiency and the need to prevent overtraining, fatigue, and patient injury.

Understanding the specific effects of different movement modes on sensory-motor cortex activity can help explore dosages (type, time, intensity, etc.) of these modes and deepen rehabilitation professionals’ understanding of exercise training mechanisms. This knowledge can guide more effectively the development of clinical rehabilitation training programs, improve their effectiveness and efficiency, and ultimately promote the development of rehabilitation therapy. In this study, we used functional near-infrared spectroscopy (fNIRS) to monitor changes in brain function during different movement patterns. This non-invasive brain imaging tool provides real-time information on cortical excitability changes caused by different movement tasks and has received increasing attention in recent years (Sangani et al., 2015). Importantly, we sought to identify patterns from fNIRS assessment results to develop evidence-based parameters and plans for exercise therapy, ultimately guiding more effective clinical treatment.

## 2 Materials and methods

This study recruited 20 healthy participants (11 males and 9 females), with a mean age of 52.38 (range: 37–78 years old) from Yueyang Hospital affiliated with Shanghai University of Traditional Chinese Medicine. All subjects were evaluated using the Edinburgh Handedness Inventory and were identified as right-handed. They had no history of neurological, physical, or psychiatric disorders. They did not take any medications that could affect it and avoided consuming coffee, tea, or other foods that could lower the cortical excitability threshold before the tests. They were fully informed about the study procedures and provided written consent to participate. The study was approved by the Human Ethics Committee of Shanghai University of Traditional Chinese Medicine (2023–132) and registered with the Chinese Clinical Trial Registry (ChiCTR2300072924).

### 2.1 fNIRS and upper limb robot

**Figure 1:**
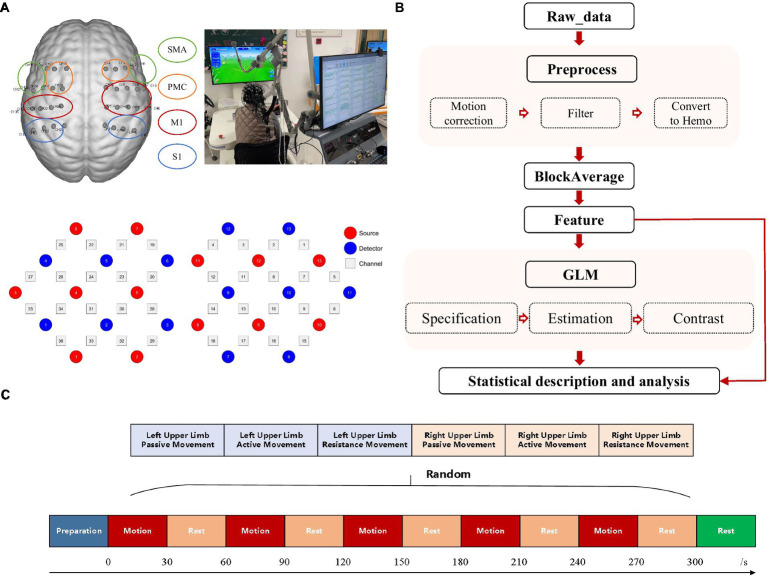
Experimental mode diagram. **(A)** Configuration of fNIRS channels and corresponding ROIs including the SMA, PMC, M1, and S1. **(B)** fNIRS data processing procedures. **(C)** Research paradigm. Subjects were randomly assigned to perform each of the three exercise modes (passive, active, resistance) with both left and right hands. Each mode lasted for 30 s, followed by a 30-s rest, and repeated five times.

Our study employed a functional near-infrared spectroscopy system (NirScan-8000A, Jiangsu Hui Chuang Medical Equipment Co., Ltd., Danyang, China) to continuously monitor cortical activity. This system utilizes continuous-wave light-emitting diodes (LEDs) at wavelengths of 730 and 850 nm, with a sampling rate of 11 Hz. For this purpose, a 36-channel system was created by configuring 26 NIRS optodes (13 sources and 13 detectors) in a rectangular format (Figure 1A). These optodes were securely mounted within a specialized cap. To ensure precise localization and consistency across measurements, a three-dimensional digitizer (Patriot, Polhemus, USA) precisely recorded the 3D coordinates of each optode and channel. Additionally, after cap placement, the subject’s scalp surface was carefully measured along specific anatomical landmarks: from the nasal root to the occipital protuberance and across the line connecting the left and right preauricular fossae. This ensured proper alignment of the electrode Cz position on the cap with the corresponding anatomical location. The regions of interest (ROIs) selected were based on Brodmann’s areas, encompassing the primary somatosensory cortex (S1), primary motor cortex (M1), premotor cortex (PMC), and supplementary motor area (SMA). The arrangement of the optodes relative to these ROIs is illustrated in Figure 1A.

This study employed the ArmGuider-S, a horizontal plane upper limb rehabilitation robot developed by Shanghai Zhuodao Medical Technology Co., Ltd., Shanghai, China (Figure 1B), to assess passive, active, and resistance movement modes in each subject. The robot comprises a working platform, linkages, and a display screen. The linkages transmit driving force from the motor to the end effector, which interacts with the subject and relays force measurements. The robot quantifies motion parameters, maintains consistent movement speed and force, and provides feedback in all three modes. Subjects completed a “butterfly catching” task by controlling the end effector along a predetermined route displayed on the screen (Figure 1C). The task trajectory and speed were pre-set for all subjects. During passive motion, the end effector automatically followed and caught butterflies at a constant speed of 0.2 m/s. In the active mode, subjects actively controlled the end effector to catch the butterflies. During resistance mode, subjects actively resisted a constant 8N force while completing the task. The butterflies moved at a fixed speed and trajectory, requiring subjects to maintain the same speed and trajectory across all exercise modalities, ensuring consistency in movement patterns.

### 2.2 Study protocol

In this study, an upper limb exercise task was administered, spanning a total duration of 330 s and segmented into five blocks. Each block comprised alternating periods of 30-s exertion and 30-s rest. Once all blocks were completed, a supplementary 30-s rest interval was incorporated to facilitate the return of the brain cortex to a state of repose. The participants engaged in three movement modalities—passive, active, and resistance—for both their left and right upper limbs, thus performing a total of six tasks: passive left-hand movement (PL), active left-hand movement (AL), resistance left-hand movement (RL), passive right-hand movement (PR), active right-hand movement (AR), and resistance right-hand movement (RR). The sequence of these tasks was randomly determined, as depicted in Figure 1C. Participants sat comfortably with their forearms secured to the support component of an upper limb robot. Before the experiment, the evaluator guided them through the robot’s operation and the experimental procedure. During the experiment, participants were instructed to relax their bodies as much as possible, minimize facial and head movements, and only move the arm being tested. They followed instructions such as “move the left hand,” “move the right hand,” and “rest” to complete the tasks in sequence. In the passive movement mode, participants relaxed their upper limbs and followed the movements of the end effector. In the active movement mode, they actively engaged and completed the training tasks under guidance. In the resistance movement mode, they needed to exert force to push the end effector while keeping their trunk and other body parts still. During the rest period, the participants will be prompted to keep their head, trunk, and limbs as still as possible, avoid talking and making facial expressions, and maintain steady breathing.

Before beginning the task, the evaluator placed the optode cap on the participant’s head, aligning its center with the intersection point of the lines connecting the ears and the nasion-inion midpoint (Cz point). Each optode’s contact pressure was adjusted to ensure good fNIRS signal quality for all channels. Once the fNIRS signal was stable, the experiment began. During the experiment, one evaluator operated the upper limb robot to ensure the participant’s minimal movement, while another monitored real-time changes in cortical activity using the fNIRS system.

### 2.3 Data analysis

Prior to data processing, it is essential to conduct data quality control. The quality control of the data will be performed using FC-NIRS software. Initially, blank channels in the raw data will be identified and excluded. Subsequently, data with clear identification of heart rate waves on the spectrum and without uncorrectable artifacts will be selected for further analysis. The data will undergo additional processing using Nirspark software. As shown in Figure 1B, the raw optical data will be converted into changes in blood oxygen concentration based on the modified Beer–Lambert law. The data will then undergo preprocessing, which includes motion correction using channel and time-range varying parameters (STD: 6, windows percent: 0.3) and bandpass filtering (0.01–0.1). The preprocessed data will be block-averaged, with block intervals set from 5 s before the start of the task to the end of a single block. The block-averaged data will be utilized for the direct computation of features such as Mean, Slope, and Difference. Additionally, these data will be further employed in the estimation of the Generalized Linear Model (GLM). Pre-processing included Gaussian smoothing with a 4-s full width at half maximum (FWHM) to correct for short-term serial correlation and a discrete cosine transform (DCT) with a 128-s period length to remove long-term trends. These parameters were incorporated into a general linear model (GLM) based on experimental conditions, with ΔOxyHb as the target variable. The GLM was estimated using a statistical parametric mapping (SPM) algorithm. Based on β values and residuals, a one-tailed t-test was used for statistical inference. A significance level of *p* < 0.05 indicated whether ΔOxyHb changes, reflecting the hemodynamic response amplitude in a specific channel, were significantly activated under specific conditions. All results will undergo FDR correction. Confirmed by the normality test, the distribution of β values does not conform to the normal distribution. Therefore, percentiles are used to represent the distribution characteristics of the data, and chi-square tests are used to compare the differences between groups. Due to the potential presence of negative β values, a modified formula for debiasing was employed to ensure the denominator’s independence. This involved calculating the absolute difference between the left and right β values as the denominator. The formula for calculating the lateralization index we use is (Lβ-Rβ)/ABS (Lβ-Rβ).

Further statistical description and analysis of eigenvalues and β values will be conducted using GraphPad software. Analysis revealed that both eigenvalues and group-level β values do not follow a normal distribution, hence percentiles and violin plots will be utilized for visualization. Wilcoxon rank-sum test will be employed for comparing differences between two groups. For comparing differences among multiple groups, Kruskal-Wallis H test will be used followed by post-hoc analysis using SNK test. Spearman correlation analysis will be applied to assess the correlation coefficients between variables.

## 3 Results

### 3.1 Demographic and clinical characteristics

This study included a total of 20 healthy right-handed subjects (11 males and 9 females) with a mean age of 52.38 years. Among them, 11 individuals had comorbidities such as diabetes (*n* = 5), hypertension (*n* = 4), or both conditions (*n* = 2). No adverse reactions occurred during the fNIRS study.

### 3.2 Cortical activation areas for different movement patterns

**Figure 2:**
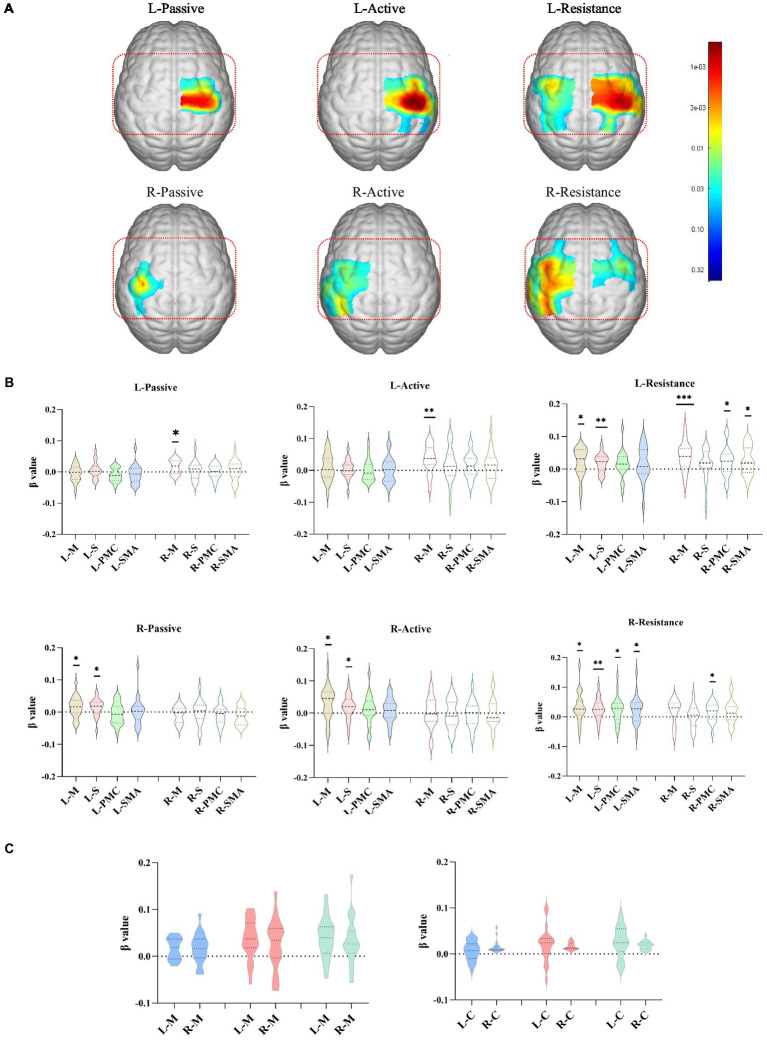
Cortical activation corresponding to different exercise types. **(A)** The cortical activation areas (GLM, *p* < 0.05, no-corrected, color bar is *p* value range from 0.005 to 0.44) in the left and right upper limbs during the execution of different exercise training. **(B)** The meanβvalue about channels in difference areas during the execution of different exercise training. **(C)** The meanβvalue about channels in motor areas and hemicortex areas during the execution of left and right upper limbs exercise trainings. *p < 0.05, **p < 0.01, ***p < 0.001. L/R-Active, active movement of left/right upper limb; L/R-Pasive, passive movement of left/right upper limb; L/R-Resistance, resistance movement of left/right upper limb; L/R-M, left/right motor area; L/R-S, left/right sensory area; L/R-PMC, left/right premotor cortex; L/R-SMA, left/right supplementary motor area; L/R-C, left/right hemicortex area.

As shown in Figure 2A, contralateral activation occurred for the three movement patterns. Passive movements mainly activated contralateral motor areas, with right passive movements activating some sensory areas, but there was no statistical difference in the mean activation of the entire sensory area. Active movement not only activated the contralateral M1, but also activated the S1. Especially when the right limb actively moved, the contralateral sensory area is generally activated (*p* < 0.05). Resistance exercise not only caused widespread and strong activation of the contralateral sensorimotor cortex (*p* < 0.01), but also caused activation of the ipsilateral PMC and SMA (*p* < 0.05) (Figure 2B). There was no difference in activation intensity between the left and right upper limbs, either in motor areas or in the entire hemicortex (*p* > 0.05; Figure 2C).

### 3.3 Comparison of activated cortex in different training modes of exercise

**Figure 3:**
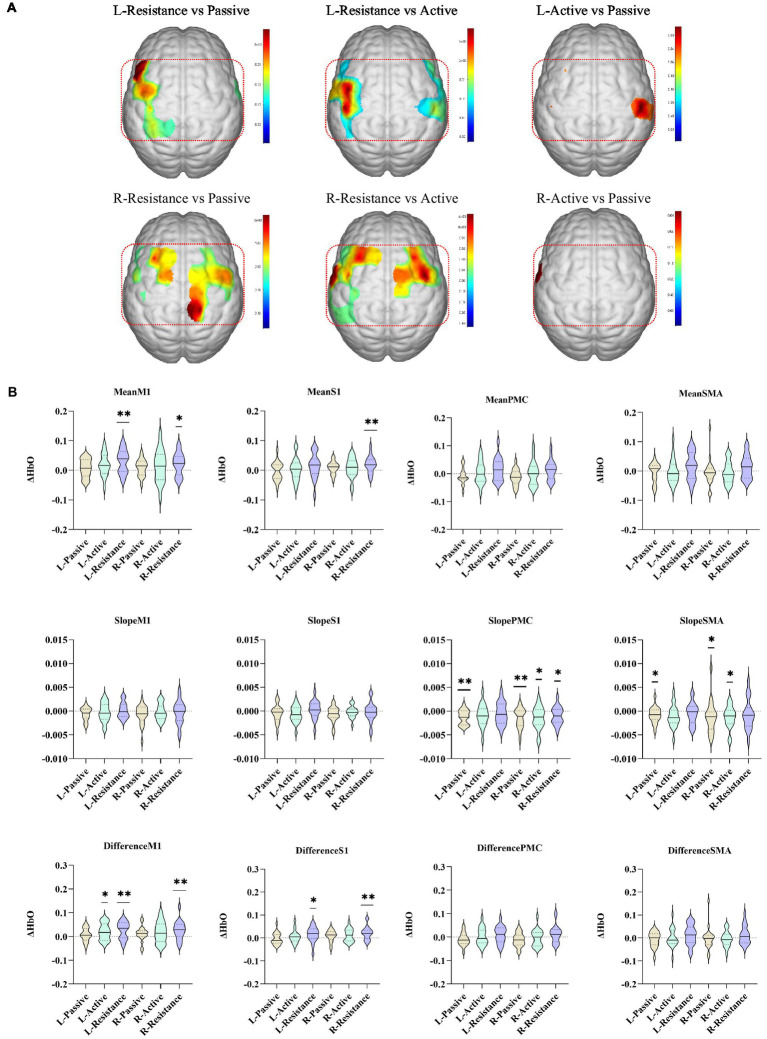
Comparison of cortex activated by different movement modes. **(A)** The colors in the figure are the p values of the corresponding channels (brain regions) (*p* < 0.05, uncorrected). The red oval area in the figure is the ipsilateral upper limb sensorimotor-related cortex. **(B)** Characteristic values of different brain regions during passive, active, and resistance exercises. *p < 0.05, **p < 0.01, ***p < 0.001. L/R-Active, active movement of left/right upper limb; L/R-Pasive, passive movement of left/right upper limb; L/R-Resistance, resistance movement of left/right upper limb.

Compared with active movement, resistance movement had a stronger effect on activating the contralateral cortex (*p* < 0.05, uncorrected). Compared with passive movement, resistance movement significantly increased the excitability of the ipsilateral M1, SMA and the contralateral S1, SMA, and M1 (*p* < 0.05, uncorrected). There was no difference in cortical activation between active and passive movement, except for individual channels (CH27: *p* = 0.03, CH6: *p* = 0.02, uncorrected) (Figure 3).

### 3.4 Lateral control of different motor modes

**Figure 4:**
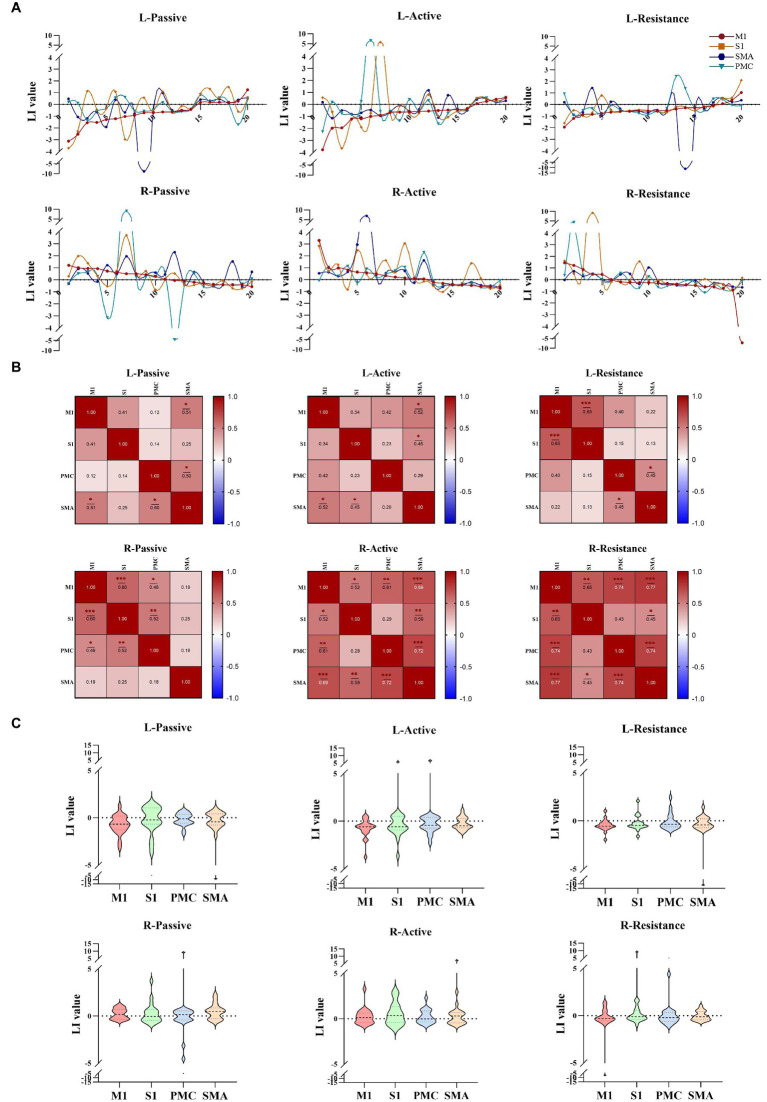
Lateralization of different movement modes. **(A)** The lateralization trends during different movement patterns of the left and right upper limbs in cortical regions (M1, S1, PMC, SMA). **(B)** The correlation between different cortical lateralization. **(C)** Lateralization values of different brain regions during different tasks. *p < 0.05, **p < 0.01, ***p < 0.001. M1, Primary motor cortex; S1, Primary somato-sensory cortex; PMC, Premotor cortex; SMA, Supplementary motor area.

The lateralization results indicated that the lateralization patterns of M1, S1, SMA, and PMC were not entirely consistent (Figure 4A). There are differences in the correlation between lateralization of different brain regions during left and right limb movements, with lower correlation between the cortex during left and right limb movements. There is a strong correlation between M1 and S1 lateralization during left resistance movement (*r* = 0.65, *p* < 0.001); When performing passive movement on the right side, the correlation between S1 and M1 lateralization is significant (*r* = 0.60, *p* < 0.001). In active exercise, the statistical results of the correlation between SMA, PMC, and M1 all showed significant differences. Especially during resistance exercise, except for PMC (*r* = 0.43, *p* > 0.05), lateralization in other brain regions was significantly correlated (Figure 4B). The degree of cortical lateralization dispersion varies when performing different motor tasks. The lateralization dispersion of passive and active movements is relatively high, while the lateralization of resistance movements is more concentrated (Figure 4C).

## 4 Discussion

This study aimed to explore the differences in sensory-motor cortex activation under different movement modes to explore the application of exercise mode dosage in rehabilitation training. By doing so, the study seeks to guide the development of exercise training programs in clinical practice, ultimately aiming to improve the effectiveness and efficiency of rehabilitation training. By using fNIRS to monitor the differences in hemoglobin content and hemodynamic parameters when healthy subjects complete passive, active, and resistance movement paradigms for the left and right upper limbs, we found that different movement modes distinctly activate the sensory-motor cortex. In terms of the size and intensity of the activated areas, resistance movement is the most significant, followed by active movement, and passive movement is the lowest. In addition, in terms of the duration of activation, resistance and active movement could activate the sensory-motor-related cortex more quickly, while the activation speed of passive movement is slower.

Rehabilitation therapy often employs passive, active, and resistance exercises tailored to the patient’s condition and goals (Damush et al., 2007). Passive training caused by peripheral limb movement can be used to relieve pain (Onishi, 2018) and induce movement (Chiyohara et al., 2020). Its activation of the motor cortex primarily depends on modulating sensory input (Nasrallah et al., 2019). Unlike active training, it engages fewer brain regions, leading to lower activation intensity (Jalalvandi et al., 2019). Choudhri et al.’s fMRI study investigated the neural basis of passive movement, demonstrating activation of the motor cortex even in an anesthetized state (Choudhri et al., 2015). This finding confirms that passive movement activates S1 through structural conduction, not visual feedback mechanisms like mirror neurons or motor imagery. This implies that when applied to conditions requiring central nervous system reshaping, such as stroke-induced hemiplegia, passive training can maintain sensorimotor cortex function and mobilize the entire sensorimotor loop, potentially facilitating movement.

Active training, the most commonly used exercise mode, is believed to activate similar brain areas as passive exercise but with greater intensity (Pittaccio et al., 2013; Estévez et al., 2014), as our research findings support. Lotze et al. suggested that active exercise emphasizes cognitive function compared to passive training, leading to significantly higher activation by engaging cortical networks (Lotze, 2003). This process involves the prefrontal cortex for setting goals, making decisions, and providing motivation, while coordinating with the parietal lobe and other areas to improve motor performance (Cao et al., 2018). Zheng et al. found that active training using upper limb robots at different speeds activated the dorsolateral prefrontal cortex (DLPFC) in addition to the somatosensory motor area (Zheng et al., 2021). Compared to simple hand movement tasks used in fNIRS studies, active training with upper limb rehabilitation robots activates more sensorimotor cortices beyond M1, including S1, SMA, and PMC (Patel et al., 2006; Fu et al., 2015). Our findings support this, demonstrating that active exercise activates a larger area of the sensorimotor cortex compared to passive exercise, encompassing contralateral S1, M1, PMC, and SMA, with higher activation intensity. This is likely due to the task-oriented nature of robot-assisted training, which requires coordination and cooperation of more brain areas. In stroke rehabilitation, active training is often combined with other methods like constraint-induced movement therapy, brain-computer interfaces (BCIs), and VR technology. These combinations aim to enable patients to consciously perform a large number of task-oriented exercises, promoting the acquisition and retention of motor skills (Škarabot et al., 2021).

Limited research exists on the specific effects of resistance training on cortical excitability, despite its common use in rehabilitation and sports training. While studies by Glover and Baker (2020) and Zheng et al. (2021) explored the impact of robot-assisted movements on cortical excitability, theirs did not directly investigate resistance exercise. Additionally, Shi et al. (2021) explored differences between resistance and non-resistance training, finding higher contralateral motor cortex activation with resistance. However, their study also lacked a comprehensive analysis of resistance training’s specific activation patterns. Previous transcranial magnetic stimulation (TMS) studies support our results, demonstrating that resistance training reduces motor cortex inhibition, leading to increased activation area and level (Siddique et al., 2020). This aligns with our findings that resistance training engages the most brain areas and exhibits the highest activation intensity compared to voluntary exercise. Our research in this area of exercise patterns presents a novel finding: the activation of the ipsilateral sensory-motor cortex during resistance movement, in addition to the contralateral side. This could be explained by changes in the corticospinal and intracortical inhibition network due to strength training. It is widely thought that beyond activating the motor cortex and intracortical circuits, the reticulospinal tract might influence unilateral or bilateral movement performance through altered synaptic efficiency between it and motor neurons or interneurons (Glover and Baker, 2020). Compared to voluntary training, resistance training activated the PMC and SMA more significantly. This could be attributed to the exercise’s greater muscle stimulation, increased demand for motor control, and a higher level of subjective involvement (Škarabot et al., 2021).

This study identified a novel finding: the lateralization consistency of the left upper limb was consistently higher than that of the right across all training modes. However, no significant difference was observed in the activation intensity between the left and right upper limbs. This suggests that the functional disparity between the left and right upper limbs may not be attributed to the function of individual brain areas, but rather to the consistency of brain region activation. Notably, no prior research has reported this phenomenon, with existing studies primarily focusing on activation differences in the dominant (right) hand during various tasks. This highlights the need for increased attention to handedness in research on hemispatial neglect. Furthermore, considering the observed differences between limbs could lead to the development of more effective upper limb rehabilitation programs in clinical settings. Bilateral brain asymmetry exists objectively, which has been confirmed in studies related to language, hearing, spatial and mathematical abilities (Marie et al., 2018; Ocklenburg and Güntürkün, 2022). In motor control, it is generally believed that the left hemisphere has an advantage in complex movements, while the right hemisphere is stronger in the spatial organization of movement patterns (Hanna-Pladdy, 2002; Mutha et al., 2012; Flores-Medina et al., 2014). Current research on motor cortex lateralization predominantly emphasizes the control advantages of the left and right hemispheres for distinct tasks. However, there is a paucity of studies investigating the differential control of identical tasks by the two hemispheres. One plausible explanation for this gap is the absence of significant differences in excitability levels between the bilateral hemispheres when performing the same task. Instead, the primary distinction appears to lie in the coordination within the movement-related cortices of each hemisphere. The functional difference between the left and right brains plays an important role in rehabilitation medicine. Schaefer et al. studied patients with left-brain stroke and right-brain stroke using the affected limb to rotate 30°. The results found that patients with left limb movement impairment completed the degree and accuracy are worse than those of patients with right limb dysfunction (Sainburg, 2002). This can be explained by the conclusion of this study: the right side of the brain needs to mobilize the participation of the S1, PMC, SMA and other cortices when performing motor control, while the left side of the brain only requires fewer brain areas to participate in coordination when performing the same function. On this basis, the same infarcted area may have a greater impact on the right side of the brain.

This study investigated the activation patterns of the sensory-motor cortex in healthy subjects during various movement modes, providing insights potentially applicable to training plans for both healthy individuals and stroke patients. Existing research suggests minimal differences in activated cortical regions between stroke patients and healthy subjects during active and passive movements (Xia et al., 2022; Li et al., 2023), supporting the potential application of our findings to rehabilitation settings.

Competition inhibition, a key theory in rehabilitation (Kwakkel et al., 2015), guides the development of rehabilitation plans for patients with varying functional states. From the perspective of the onset time of stroke, restricted limb movement often necessitates passive exercise to preserve somatosensory-motor cortex function on the affected side in the early stage of stroke (Lee et al., 2018). Additionally, low-frequency TMS is sometimes applied to the healthy side’s somatosensory-motor cortex to prevent over-excitation (Blesneag et al., 2015). During the course of patient recovery, with demonstrably improved motor function, a multimodal approach incorporating both passive and active training modalities is frequently implemented. Passive exercise at this stage can activate the spinal cord loop and reduce muscle tone (Yang et al., 2017). Active training, particularly task-oriented training, is highly recommended in stroke rehabilitation due to its versatility and ability to maximize excitation in the affected somatosensory-motor cortex and relevant cognitive areas (Jeon et al., 2015). However, regaining upper limb function often requires inhibiting the healthy side to increase affected side excitability. While early resistance exercise may activate both sides of the cortex (further research is needed to assess its impact on treatment outcomes), its suitability remains unclear. Additionally, it is important to investigate whether resistance training following low-frequency TMS on the healthy side diminishes the inhibitory effect. In comparison, non-resistant active training does not significantly increase healthy-side excitability, potentially making it a more suitable option. Based on these considerations, resistance training might not be optimal for the functional reshaping stage, potentially being more beneficial after motor skills have been acquired and retained. On the other hand, from the perspective of stroke upper limb function, taking Brunnstrom upper limb function stages as an example: Stage I emphasizes maintaining sensorimotor loop function with passive movement and peripheral-central magnetic stimulation. Stage II shifts to promoting motor evocation and learning through combined active and passive training, potentially aided by low-frequency contralateral transcranial magnetic stimulation to avoid over-excitability in the unaffected side. Stage III focuses on managing spasticity with continued passive training for tension reduction, increased active training targeting extensor muscles, and avoiding resistance training, possibly with additional support from peripheral-central magnetic stimulation. Stages IV-V prioritize promoting motor learning through active training, including the gradual introduction of resistance training for extensor muscles. Finally, Stage VI, as function nears normal, transitions to resistance and active training focusing on functional tasks and real-world application.

This study conducted a relatively detailed demonstration of different upper limb movement patterns, compensating for the lack of movement pattern types and the insufficiency in exploring the trend of activation lateralization in previous research. The results of this study have implications for future motor rehabilitation of neurological diseases, especially stroke. However, this study also has certain limitations. The small sample size of subjects included in this study is not conducive to a more comprehensive summary of the characteristics of motor pattern activation. Additionally, this study should have included patients with neurological diseases (stroke) as subjects to better interpret the differences in cortical activation between the two groups when completing different movement patterns and to guide exercise prescriptions for neural rehabilitation. This aspect will be improved in our subsequent research.

## 5 Conclusion

Passive movement, active movement, and resistance movement all activate the contralateral motor cortex. Among them, resistance exercise activates the most cortex, and PMC plays an important role in resistance exercise. Passive movement does not significantly activate the sensory cortex, and movement modes such as active and resistance that require more patient participation have an effect on the sensory area. Furthermore, the left cortex showed weaker intercortical coherence in controlling movement, while the right cortex showed stronger coherence.

## Data availability statement

The raw data supporting the conclusions of this article will be made available by the authors, without undue reservation.

## Ethics statement

The studies involving humans were approved by the Ethics Committee of Shanghai University of Traditional Chinese Medicine Affiliated Yueyang Hospital of Integrated Traditional Chinese and Western Medicine. The studies were conducted in accordance with the local legislation and institutional requirements. The participants provided their written informed consent to participate in this study. Written informed consent was obtained from the individual(s) for the publication of any potentially identifiable images or data included in this article.

## Author contributions

WL: Conceptualization, Data curation, Resources, Writing – original draft. GGZ: Conceptualization, Methodology, Visualization, Writing – original draft. YJ: Formal analysis, Software, Writing – original draft. CM: Project administration, Writing – original draft. GHZ: Supervision, Writing – review & editing. DX: Supervision, Validation, Writing – review & editing.
